# Laparoscopic partial nephrectomy for tumors 7cm and above. Perioperative outcomes

**DOI:** 10.1590/S1677-5538.IBJU.2016.0642

**Published:** 2017

**Authors:** Matvey Tsivian, Efrat Tsivian, Yury Stanevsky, Roman Bass, A. Ami Sidi, Alexander Tsivian

**Affiliations:** 1 Division of Urology and Department of Surgery, Duke University Medical Center Durham, NC; 2 Department of Urologic Surgery, The E. Wolfson Medical Center, Holon and Sackler School of Medicine Tel Aviv University, Tel Aviv, Israel

**Keywords:** Nephrectomy, Laparoscopy, Neoplasms

## Abstract

**Purpose:**

To assess and report the outcomes of laparoscopic partial nephrectomy) LPN) for T2 renal masses.

**Materials and Methods:**

Retrospective review of patients undergoing LPN for clinically localized renal masses ≥7cm between the years 2005-2016. Descriptive analyses were generated for demographics, lesion characteristics, perioperative variables (operative time, warm ischemia time (WIT), estimated blood loss (EBL), intra-operative and postoperative complications (IOC and POC) and pathologic variables (pathology, subtype and Fuhrman grade).

**Results:**

A total of 27 patients underwent LPN for a T2 renal mass at our institution between 2005 and early 2016 of which 19 were males. The mean age was 66 (52-72). All procedures were transperitoneal with 16 on the right and 11 on the left. Median operative time was 200 minutes (IQR 181-236) and median WIT 19 minutes (IQR 16-23). EBL was 125mL (IQR 75-175). One case was converted to laparoscopic radical nephrectomy due to suspected tumor thrombus in the renal vein. Surgical margins were positive in one renal tumor in a patient with multiple tumors. There was a total of 2 IOC (7.4%) and 3 POC (11%) classified as Clavien grade 3.

**Conclusions:**

To our knowledge, this series is the first to describe the outcomes of LPN for cT2 renal masses. In our series, LPN for larger renal masses appears feasible with favorable perioperative outcomes. Additional data are needed to further explore the benefits of minimally invasive surgical approaches to larger renal masses.

## INTRODUCTION

Laparoscopic partial nephrectomy (LPN) is a valid treatment option for small renal masses (SRM) (1, 2). It has been shown that LPN provides comparable overall survival (OS), cancer specific survival (CSS) and progression free survival (PFS) when compared to open partial nephrectomy (3-5) while maintaining the benefits of nephron-sparing surgery (NSS) in preservation of renal parenchyma and therefore decreased risk of chronic kidney disease.

Studies have described positive outcomes with LPN for stage T1 masses (4-6). Only scarce data exist, however, on the use of Laparoscopy in NSS for T2 masses (7, 8). While in the widespread of robotics, mainly in the United States has largely replaced the use of pure laparoscopy, in other parts of the world, laparoscopy remains the mainstay of minimally invasive surgery and an extremely relevant topic for discussion. At our institution, the standard of care for renal masses is LPN. We have previously reported on our outcomes for tumors larger than T1a (7). Herein, we analyze the outcomes of LPN for tumors of 7cm or larger.

## PATIENTS AND METHODS

After approval from our Institutional Review Board we retrospectively reviewed our prospectively collected database to identify patients undergoing LPN for a clinical T2 renal mass. Clinical stage was determined on cross sectional imaging. A total of 27 patients underwent LPN for renal masses ≥7cm between 2005 and early 2016. All surgeries were performed by a single surgeon (AT). The variables that were examined when reviewing our database were demographics (age, gender), lesion characteristics: side, centrality, location and size. Perioperative variables collected included: operative time (OT), warm ischemia time (WIT), estimated blood loss (EBL), concomitant surgery, conversion to laparoscopic radical nephrectomy (RN) or to open surgery, intra-operative complications (IOC) and post-operative complications (POC). POC were classified according the modified Clavien system (9). Pathologic variables included malignant vs. benign pathology, subtype and Fuhrman grade when applicable. Descriptive analyses were generated. Data are reported as median (interquartile range, IQR) or number (%).

### Surgical technique

Our surgical technique has evolved over the years. We use the standard transperitoneal approach with three to four trocars as previously described (10). After dissecting the renal vessels and identifying the tumor, the anticipated resection margins are marked with cautery under ultrasound guidance. Typically, a bulldog clamp is applied to the renal artery only, without venous clamping. In cases of central tumors whereby venous clamping is deemed beneficial, a single bulldog clamp is applied to artery and vein en bloc. The mass is excised and trapped in a bag. The defect is then closed in two layers. A running suture secured on both ends with an absorbable clip controls the resection bed (regardless of whether the collecting system is violated) and for the superficial layer a running suture interrupted by absorbable clips on one side of the defect is used (11). With the evolution of our technique we do not routinely use bolsters or other additional hemostatic agents aside from sutures. Routinely, ≥10mm ports sites are closed using the Endoclose TM device (United States Surgical, Norwalk, CT).

## RESULTS

A total of 27 patients underwent LPN for T2 (7-15cm) renal masses at our institution between 2005 and 2016. Patient characteristics are detailed in Table-1. The median age was 66 (IQR 52-78) with male predominance (70.4%). The median radiographic tumor size was 80mm (IQR 72-110). In 2 (7.4%) cases lesions were bilateral. Most of the lesions were central (46.2%) followed by hilar (38.7%) and peripheral (11.5%). Posterior lesions accounted for 53.8% of cases and the upper pole was involved in 53.8% of cases. The majority of masses (63%) were malignant with predominance of the clear cell subtype. All 27 cases were managed laparoscopically with a median operative time of 200 minutes (IQR 180-233) and WIT of 19 minutes (IQR 16-23). Estimated blood loss was 125mL (IQR 88-163). One case was converted to laparoscopic radical nephrectomy due to suspected tumor thrombus in the renal vein. Surgical margins were positive in one renal tumor in a patient with multiple tumors. There was a total of 2 intraoperative complications including a renal vein injury that was repaired intraoperatively and a slipped bulldog clamp resulting in bleeding requiring transfusion. There were 3 postoperative complications, classified as Clavien grade 3: a ureteral injury that was identified postoperatively requiring open ureteroureterostomy on postoperative day 2, urinoma due to obstructing ureteral stone 2 weeks after surgery, requiring stent placement and a delayed bleeding 7 days following the procedure that was managed with angioembolization.

**Table 1:** Cohort characteristics.

Variable		Number (%)/Median (IQR)
Number of patients		27
Age, years		66 (52-78)
**Gender**		
	Female	8(29.6%)
	Male	19(70.4%)
**Side**		
	Right	16(59.3%)
	Left	11(40.7%)
Bilateral		2(7.4%)
Concomitant surgery		3(11%)
**Centrality**		
	Central	13(46.2%)
	Hilar	10(38.7%)
	Peripheral	3(11.5%)
Location*		
	Anterior	9(34.6%)
	Medial	2(7.6%)
	Lateral	1(3.8%)
	Posterior	14(53.8%)
**Pole location**		
	Low	8(30.8%)
	Mid	4(15.4%)
	Upper	14(53.2%)
Tumor size, mm		80(72-110)
OT, minutes		200(181-236)
WIT, minutes		19(16-23)
EBL, mL		150(75-175)
IOC		2(7.4%)
POC		3(11.1%)
Conversion to radical		1(3.7%)
**Malignancy**		
	Benign	10(37%)
	Malignant	17(63%)
Positive margins		1(3.7%)
**Subtype** **		
	Clear cell	7(41.2%)
	Chromophobe	3(17.6%)
	Cystic	2(11.8%)
	Papillary	5(29.4%)
**Fuhrman**		
	I	4
	II	4
	III	5
	IV	0

* One case of pelvic kidney, therefore centrality, location and pole location could not be classified.

** percentage out of malignant masses

**T=**operative time; **WIT=**Warm ischemia time; **EBL=**Estimated blood loss; **IOC=**Intraoperative complications; **POC=**Postoperative complications

**Figure 1:**
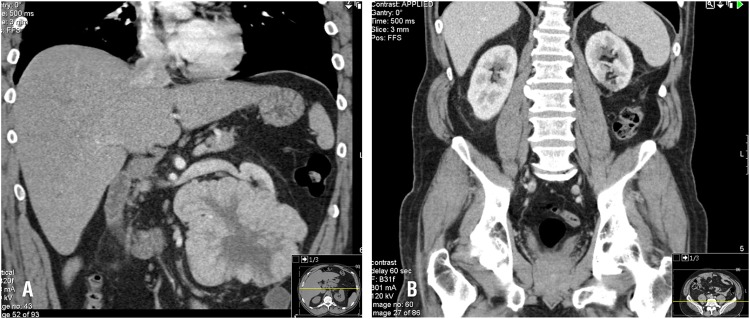
14cm tumor of the left kidney. Preoperative (A) and 6 months postoperative imaging (B).

**Figure 2:**
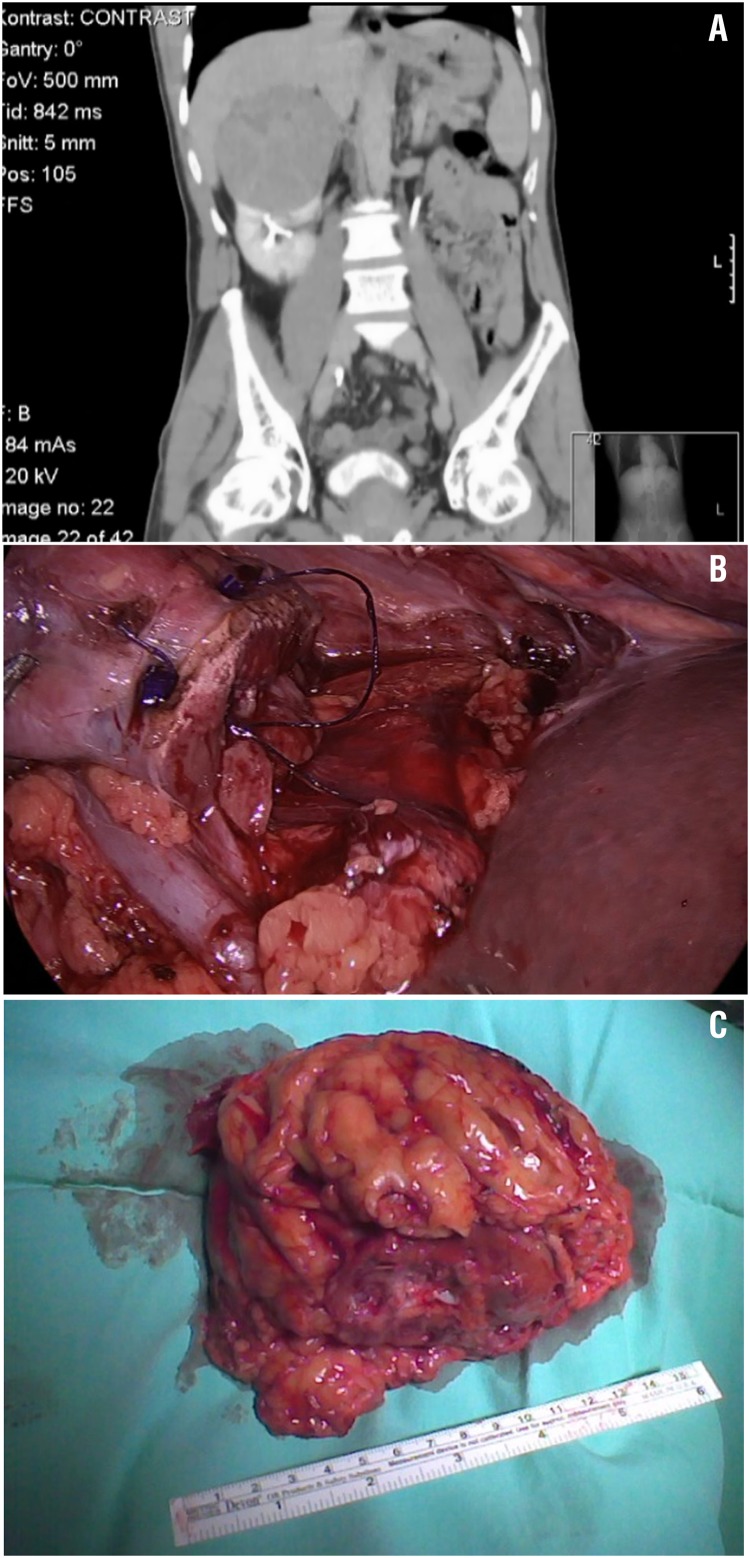
10cm right renal tumor. Preoperative imaging (A), intraoperative view of heminephrectomy crater (B) and specimen (C).

## DISCUSSION

Laparoscopic partial nephrectomy is a well-established treatment option for small renal masses that is recommended by the major guidelines for T1 renal masses (1); moreover, it has been shown to have comparable short term and long term cancer control as its open counterpart in several studies (12, 13). Although PN for renal masses ≥7cm has already been shown to be a valid alternative for RN (14, 15), evidence of LPN feasibility for larger renal masses is scarce. All published series on NSS for T2 disease reporting on a mixed surgical approach, mostly open, with very small cohorts of patients undergo laparoscopic surgery.

Currently, at our institution, LPN is the preferred management option for most patients with localized renal mass. Here we described our experience with LPN for renal tumors ≥7cm. In the present study, perioperative outcomes were encouraging with a median OT of200 minutes (181-236). This is in line with what has been previously reported on minimally invasive partial nephrectomy for large renal masses. Brandao et al. reported a similar median OT when they compared Robot-Assisted LPN (RALPN) outcomes for tumors ≥7cm and ≤4cm (16). Karellas et al. reported a median of 170 minutes on their series of 34 patients with cortical renal masses ≥7cm of which 5 were performed laparoscopically (17).

WIT under 20 minutes is considered safe to minimize renal ischemic damage (18). Our median WIT of 19 minutes is comparable to what has been reported previously for renal masses ≥4cm. In a multi-institutional comparative analysis of RALPN Petros et al. reported a median WIT of 24 minutes for masses >4cm and of 17 minutes ≤4cm (19). Ficarra et al. achieved a WIT of 22 minutes with only a minority of cases with WIT >30 minutes (20). A WIT of 38 however was described by Simmons et al. (21) which the authors attributed to a large portion of complex tumors in their series. Most of the tumors in our series had a central location (46.2% central and 7.6% central-hilar). We believe that our WIT times are in line with the literature and are favorable.

Complication rates in our series include 2 (7.4%) IOC and 3 (11%) POC of grade 3 according to the modified Clavien classification (9). Similar rates were described by Ficarra et al. (20). The same percentage of major complications (10.9%) was seen in Becker et al. series of 91 open partial nephrectomies that included only 1 had RALPN (15).

A low percentage of positive surgical margins is described in various studies of minimally invasive partial nephrectomy (19, 22, 23). We only had one patient with positive margins (5.9% out of all malignant masses). The patient had 2 masses on the upper and lower poles. The margins were positive on the smaller mass that measured 2.5cm.

Our present study has several limitations. Its retrospective, single center nature raises difficulty in the application of these results to general practice. It is also important to consider that these complex cases were done by a single experienced surgeon and therefore, our results may be challenging to replicate in a different setting. Furthermore, even though perioperative outcomes appear excellent, longer follow-up is required for our oncological outcomes to mature.

## CONCLUSIONS

To our knowledge, this series is the first to describe the outcomes of pure LPN for cT2 renal masses aside from scattered reports. In our series, LPN for larger renal masses appears feasible with favorable perioperative outcomes. Additional data are needed to further explore the benefits of minimally invasive surgical approaches to larger renal masses and determine oncological outcomes.
